# Related factors to the coping style of patients with sudden sensorineural hearing loss

**DOI:** 10.1093/inthealth/ihac046

**Published:** 2022-07-18

**Authors:** Yang Yuan, Lu Lin, Hong Wang, Congyan Xie, Qiuyun Chen, Haixia Li, Li Tian

**Affiliations:** First Affiliated Hospital of Soochow University, Suzhou 215006, China; School of Nursing, Medical College of Soochow University, Suzhou 215006, China; First Affiliated Hospital of Soochow University, Suzhou 215006, China; School of Nursing, Medical College of Soochow University, Suzhou 215006, China; Suzhou Kowloon Hospital, Shanghai Jiao Tong University School of Medicine, Suzhou 215021, China; School of Nursing, Medical College of Soochow University, Suzhou 215006, China; School of Nursing, Medical College of Soochow University, Suzhou 215006, China; Suzhou Kowloon Hospital, Shanghai Jiao Tong University School of Medicine, Suzhou 215021, China; First Affiliated Hospital of Soochow University, Suzhou 215006, China; School of Nursing, Medical College of Soochow University, Suzhou 215006, China

**Keywords:** coping style, related factors, sudden sensorineural hearing loss (SSNHL), young and middle-aged

## Abstract

**Background:**

Coping style can affect the patient's physical and mental health management. Therefore this study aimed to identify factors related to the coping style of young and middle-aged sudden sensorineural hearing loss (SSNHL) patients to provide reference for clinical nursing practice.

**Methods:**

A cross-sectional study was conducted on young and middle-aged SSNHL patients hospitalized in the otolaryngology departments of four hospitals in Suzhou City, China. A paper-based self-administered questionnaire investigated the patient's coping style and related factors. Multiple linear stepwise regression analysed the effective related factors in patients’ coping styles.

**Results:**

Among 872 patients, 866 completed the survey, with an average age of 37.27 y. Factors related to the coping style of these patients included gender, chronic diseases, history of trauma, social support and type D personality (p<0.05). Female patients adopt more negative coping styles than male patients. Patients with chronic diseases or a history of trauma had more positive coping styles. Higher social support scores were related to improvements in coping style. Patients with type D personality were more likely to adopt negative coping styles.

**Conclusions:**

This study suggests that psychological assessment of patients, chronic diseases, history of trauma, social support and type D personality may benefit the understanding of these patients’ coping styles and, as a consequence, may improve their stress management.

## Introduction

The majority of patients affected by hearing loss (>80%) suffer from sensorineural hearing loss. Apart from age-associated, drug-induced and noise-induced hearing loss, idiopathic sudden sensorineural hearing loss (SSNHL) is the most frequent cause.^1^ SSNHL refers to sensorineural hearing loss of unknown cause that occurs suddenly within 72 h and the hearing loss is ≥30 dB on at least three frequencies.^2^ In recent years, the annual incidence of this disease is 5–27 per 100 000 people, showing an upward trend, and the age of onset tends to be younger.^1,2^ The onset of this hearing loss is sudden and often accompanied by many uncomfortable symptoms, including tinnitus, dizziness and vision loss. These symptoms can affect the normal work and social activities of these patients and make the patients prone to mental and psychological disorders (such as anxiety and fear).^3^ As the mainstay of family and society, young and middle-aged patients are more likely to feel the various pressures caused by the disease, which makes the incidence of anxiety and depression in young and middle-aged SSNHL patients significantly higher than in other groups.^4^ Therefore it is imperative to pay attention to the physical and mental health of young and middle-aged SSNHL patients.

Coping style refers to individuals consciously adjusting their own aspects (emotional, cognitive, behavioural and physical) or environmental aspects to reduce stress.^5^ Coping style affects the patient's physical and mental health management and can be seen as an intermediate mechanism between health and stress.^5^ The more effective the coping style, the lower the risk of mental health problems and stressful life events.^6^ If the coping style is effective, the patient's psychological distress will be reduced; in contrast, if the coping style is inappropriate, the patient's distress will be aggravated and the patient may easily develop acute stress disorder (ASD).^7^ The results of our previous research also verified that active and effective coping styles can improve the illness experience of this patient population and reduce their psychological burden.^8^

Social support refers to the actual help, emotional support and information assistance provided by meaningful groups (such as family members, friends, colleagues, relatives and neighbours) when the individual is in a difficult situation.^9^ Several studies have shown that younger adults and people with high social pressure or low social support have worse mental health.^10,11^ Positive coping styles and family support are protective factors for psychological problems.^12^ Wlodarczyk's study^13^ indicated that the coping style of seeking social support and problem solving had a positive impact on subjective health. In the early stage of disease treatment, if the patient chooses to cope with the disease and has good social support, he/she will have greater expectations for treatment and recovery from the disease, which is more conducive to health. In addition, type D personality is closely related to symptoms such as depression, anxiety, chronic tension, pessimism, lack of perceived social support, lower subjective well-being and self-esteem, dissatisfaction with life, decreased quality of life and poor body image.^14^ One study found that type D personality is associated with more passive coping styles of resignation or withdrawal.^15^

In view of the importance of coping styles in stressful events, when these patients face tremendous pressure from study, work or family, they need to adopt active coping styles. However, in current clinical nursing practices, the medical staff pay more attention to the patient's treatment plan and effect, while ignoring changes in the patient's psychological and living conditions after the onset of disease, as well as their coping styles. Therefore this study explored factors related to the coping styles of young and middle-aged SSNHL patients, aiming to provide references for clinical nursing practice, which has important practical significance for improving the quality of life in these patients.

## Methods

### Study design

**Figure 1.**
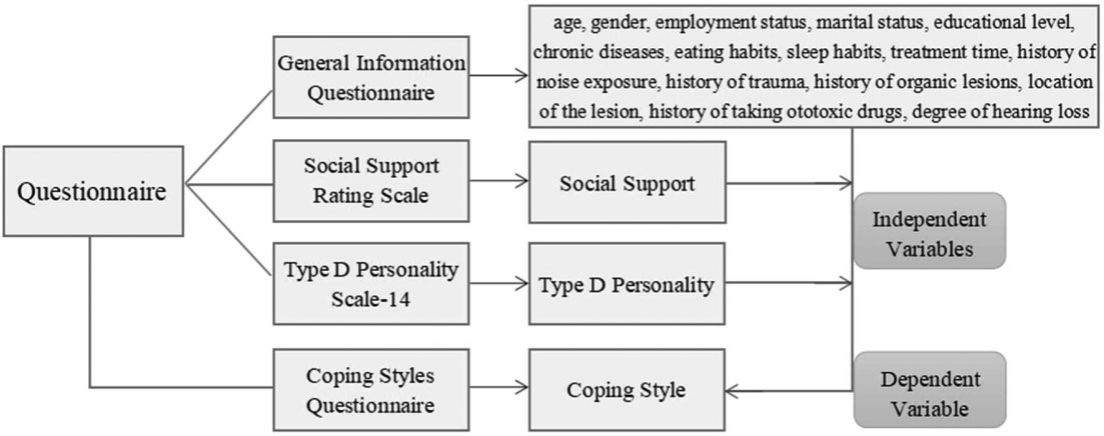
Study design.

This study used a cross-sectional design. From December 2019 to March 2021, a survey of young and middle-aged SSNHL patients in the otolaryngology departments of four level A tertiary hospitals in Suzhou, China was conducted. The inclusion criteria were as follows: inpatients who met the diagnostic criteria of SSNHL in the 2015 Guidelines for the Diagnosis and Treatment of SSNHL and were first diagnosed with SSNHL, age 18–64 y (young and middle-aged), have basic comprehension and communication skills and voluntary participation in this study. A preliminary survey questionnaire was designed according to the research purpose as well as opinions of experts, interviews with patients and relevant literature.^5–15^ The questionnaire was piloted in 15 young and middle-aged SSNHL patients and finalized after revision. In this study, the dependent variable was coping style, while the 17 independent variables included demographic variables (age, gender, employment status, marital status, education level, chronic diseases, eating habits, sleep habits, treatment time, history of noise exposure, history of trauma, history of organic lesions, location of the lesion, history of taking ototoxic drugs and degree of hearing loss) and items of two scales (social support and type D personality).^5–15^ An overview of the study design and all variables is shown in Figure 1.

### Sample size

Based on the Kendall criterion, the number of observations is at least 5–10 times the number of variables. According to Sun and Xu,^16^ the sample size can be 10–20 times the research variables. Therefore the calculated sample size of this study was 17 × 20 = 340 cases. Considering that 10% of the questionnaires might be invalid, the sample size should be no less than 378 cases. In order to make the sample more representative, a total of 872 young and middle-aged SSNHL patients were surveyed. Six of the collected questionnaires were incomplete (no answers to at least three questions) or invalid (the same answers to most items) and 866 were valid, with the effective response rate being 99.31%.

### Data collection

Three researchers were uniformly trained as investigators to collect data. The investigators identified eligible patients based on the inclusion and exclusion criteria and distributed questionnaires after obtaining the patient's written informed consent. The investigators explained the questionnaire completion method and cautions to the patients. The patients independently completed the questionnaire in an quiet environment and the questionnaire was returned on the spot. The questionnaires took 15–20 min to complete. The questionnaire consisted of four parts.

### General information questionnaire

The general information questionnaire included the patient's demographic data (age, gender, employment status, marital status, education level etc.) and clinical data (chronic diseases, history of noise exposure, history of trauma, history of organic lesions, location of the lesion, history of taking ototoxic drugs, degree of hearing loss etc.). It was filled out by the patient or obtained from the patient's medical record.

### Coping Styles Questionnaire (CSQ)

The CSQ was compiled by Jihua Xiao and used to measure individual strategies for stress events.^17^ The CSQ consists of a total of 62 items in six subscales. The six subscales are problem solving (1, 2, 3, 5, 8, 19, 29, 31, 40, 46, 51, 55), self-blame (15, 23, 25, 37, 39, 48, 50, 56, 57, 59), help seeking (10, 11, 14, 36, 39, 42, 43, 53, 60, 62), fantasy (4, 12, 17, 21, 22, 26, 28, 41, 45, 49), avoidance (7, 13, 16, 19, 24, 27, 32, 34, 35, 44, 47) and rationalization (6, 9, 18, 20, 30, 33, 38, 52, 54, 58, 61). For each item, 1 point is given when ‘yes’ is chosen and 0 when ‘no’ is chosen. There are four items with reverse scoring (19, 36, 39, 42). Problem solving, help seeking and rationalization are active and mature coping styles, while self-blame, avoidance and fantasizing are negative and immature coping styles. The score for each subscale reflects the characteristics of different types of coping behaviour (positive coping or negative coping) and higher scores represent greater coping styles. The CSQ has good reliability and validity. Jihua Xiao verified the reliability and validity of the Chinese version of the CSQ in adolescent students and neurotic patients. The results showed that the test–retest reliability of each subscale in the adolescent student group was 0.62–0.72 and that in the neurosis control group it was 0.63–0.73. The load value of each factor item was >0.35.^17^

### Social Support Rating Scale (SSRS)

The SSRS was compiled by Xiao in 1986 and revised in 1990.^18^ The scale contains 10 items in three dimensions: subjective support (1, 3, 4, 5), objective support (2, 6, 7) and utilization of social support (8, 9, 10). The total score is the sum of scores for the 10 items. The higher the score, the better the social support. The SSRS has good reliability and validity, with a test–retest reliability of 0.92 and a Cronbach’s α of 0.89–0.94.^18^

### Type D Personality Scale-14 (DS14)

The DS14 contains 14 items in two dimensions: negative affectivity (NA; 2, 4, 5, 7, 9, 12, 13) and social inhibition (SI; 1, 3, 6, 8, 10, 11, 14).^19^ The respondents rate their personality on a 5-point Likert scale ranging from 0 to 4 (0, false; 1, rather false; 2, neutral; 3, rather true; 4, true), with a maximum score of 28 in each dimension. According to clinical criteria, respondents with NA ≥10 and SI ≥10 are considered to have a type D personality.^19^

### Statistical analysis

SPSS version 24.0 (IBM, Armonk, NY, USA) was used for statistical analysis of all data. The significance level was set to 0.05 and p<0.05 was considered statistically significant. The measurement data were described by the mean and standard deviation (SD) and the counting data was described by the frequency and composition ratio. A t-test and variance analysis were used to analyse the differences in the coping style scores of young and middle-aged SSNHL patients in the general data. Pearson correlation analysis was used to analyse the correlation of patients’ coping styles with social support and type D personality. Multiple linear stepwise regression analysis was used to identify the main factors affecting the coping style of patients.

### Ethical considerations

Ethical approval to perform the study was obtained from the Ethics Committee of Soochow University, Suzhou, China (SUDA20200515H02). Written informed consent was obtained from each participant prior to the survey.

## Results

### Patient characteristics

All participants were 18–64 y of age and the average age was 37.27±10.65 y. A total of 381 were male (44%), 485 were female (56%), 724 were employed (83.6%), 712 were married (82.22%), 663 had chronic diseases (76.56%), 146 were exposed to noise (16.86%), 45 had a trauma history (5.2%) and 17 had a history of organic lesions (1.96%). The patients mainly had unilateral disease (746 [86.14%]) and the degree of hearing loss was mainly mild (319 [36.84%]). Since information on patients’ eating habits, sleeping habits and treatment time was not collected completely, these could not be included in the final statistical analysis.

### Scores for coping style, social support and type D personality

Among the coping styles of young and middle-aged SSNHL patients, the positive coping style was scored higher (the problem-solving and help-seeking dimensions). In terms of social support, subjective support scored the highest. A total score of <35 was categorized as low social support (340 people), 35–45 was categorized as moderate social support (425 people) and >45 was categorized as high social support (101 people); the overall level of social support was not high. According to the clinical criteria, patients with an NA score ≥10 and an SI score ≥10 were considered to have a type D personality. Therefore, in this study there were 356 patients (41.11%) with type D personality and 510 (58.89%) with non-D-type personality. See Table 1 for specific scores.

**Table 1.** Coping style, social support and type D personality scores of young and middle-aged SSNHL patients (N=866, ${\bar{\rm{x}}} $±s)

Variables	Score
Coping style	
Problem solving	0.82±0.18
Self-blame	0.37±0.30
Help seeking	0.64±0.21
Fantasy	0.52±0.25
Avoidance	0.50±0.24
Rationalization	0.45±0.25
Social support	
Subjective support	21.96±4.34
Objective support	8.18±2.34
Utilization of social support	6.88±1.90
Total score	37.02±6.63
Type D personality	
Negative affectivity	10.60±5.19
Social inhibition	11.63±4.90

### Univariate analysis of scores in various dimensions of coping style

The scores of young and middle-aged SSNHL patients of different genders in the problem-solving dimension were statistically significant (t=2.002, p=0.046) and male patients had higher scores than female patients; the scores of patients of different genders in the help-seeking and fantasy dimensions were statistically significant (t=−2.249, p=0.025; *t*=−2.202, p=0.028, respectively) and female patients had higher scores than male patients. The scores of young and middle-aged SSNHL patients with or without chronic diseases in the rationalization dimension were statistically significant (t=3.400, p=0.001) and patients with chronic diseases scored higher than those without chronic diseases. The scores of young and middle-aged SSNHL patients with or without a trauma history in the help-seeking, fantasy and rationalization dimensions were statistically significant (t=2.491, p=0.013; t=2.105, p=0.036; t=2.119, p=0.034, respectively) and patients with a trauma history scored higher than those without a trauma history (Table 2).

**Table 2.** Univariate analysis of scores in various dimensions of coping style in young and middle-aged SSNHL patients (N=866, ${\rm{\bar{x}}}$±s)

Variables	Value, n (%)	Problem solving	Self-blame	Help seeking	Fantasy	Avoidance	Rationalization
Gender							
Male	381 (44.00)	0.83±0.18	0.36±0.29	0.63±0.21	0.50±0.25	0.50±0.24	0.45±0.25
Female	485 (56.00)	0.81±0.19	0.38±0.30	0.66±0.29	0.54±0.24	0.50±0.25	0.45±0.25
t (p)		2.002 (0.046)	−1.400 (0.162)	−2.249 (0.025)	−2.202 (0.028)	−0.049 (0.961)	−0.224 (0.823)
Employment status							
Employed	724 (83.60)	0.82±0.18	0.37±0.29	0.64±0.21	0.52±0.25	0.50±0.24	0.50±0.22
Unemployed	142 (16.40)	0.81±0.18	0.37±0.30	0.65±0.21	0.53±0.23	0.50±0.23	0.49±0.21
t (p)		0.667 (0.505)	0.091 (0.928)	−0.652 (0.514)	−0.644 (0.519)	0.095 (0.924)	0.311 (0.756)
Marital status							
Single/never married/ Divorced/widowed	154 (17.78)	0.82±0.18	0.33±0.29	0.66±0.21	0.53±0.24	0.48±0.24	0.47±0.21
Married/cohabitating	712 (82.22)	0.82±0.19	0.37±0.30	0.64±0.21	0.52±0.25	0.50±0.24	0.50±0.22
t (p)		0.172 (0.864)	0.168 (0.867)	1.097 (0.273)	0.675 (0.500)	−1.074 (0.283)	−1.305 (0.192)
Education level							
Senior high school/technical school and below	210 (24.25)	0.80±0.19	0.38±0.31	0.63±0.21	0.54±0.26	0.52±0.24	0.51±0.23
Junior college and above	656 (75.75)	0.83±0.18	0.37±0.29	0.65±0.21	0.51±0.24	0.49±0.24	0.49±0.22
t (p)		−1.903 (0.057)	0.251 (0.802)	−0.848 (0.397)	1.275 (0.203)	1.237 (0.217)	1.287 (0.199)
Chronic diseases							
Yes	663 (76.56)	0.82±0.19	0.38±0.30	0.64±0.21	0.52±0.25	0.50±0.25	0.47±0.24
No	203 (23.44)	0.82±0.17	0.35±0.28	0.64±0.20	0.51±0.25	0.51±0.24	0.40±0.27
t (p)		0.194 (0.846)	0.955 (0.340)	−0.075 (0.940)	0.753 (0.451)	−0.517 (0.605)	3.400 (0.001)
Exposure to noise environment							
Yes	146 (16.86)	0.83±0.16	0.38±0.32	0.62±0.18	0.51±0.28	0.53±0.25	0.42±0.28
No	720 (83.14)	0.82±0.19	0.37±0.29	0.65±0.21	0.52±0.24	0.49±0.24	0.46±0.24
t (p)		0.703 (0.482)	0.208 (0.835)	−1.243 (0.214)	−0.768 (0.443)	1.467 (0.143)	−1.705 (0.089)
History of trauma							
Yes	45 (5.20)	0.84±0.14	0.42±0.34	0.72±0.20	0.60±0.25	0.56±0.23	0.53±0.27
No	821 (94.80)	0.82±0.19	0.37±0.29	0.64±0.20	0.52±0.25	0.50±0.24	0.45±0.25
t (p)		0.892 (0.373)	1.064 (0.288)	2.491 (0.013)	2.105 (0.036)	1.720 (0.086)	2.119 (0.034)
History of organic lesions							
Yes	17 (1.96)	0.87±0.14	0.36±0.33	0.62±0.22	0.54±0.26	0.54±0.25	0.48±0.28
No	849 (98.04)	0.82±0.19	0.37±0.30	0.64±0.20	0.52±0.25	0.50±0.24	0.45±0.25
t (p)		1.141 (0.254)	−0.186 (0.853)	−0.413 (0.680)	0.357 (0.721)	0.615 (0.538)	0.418 (0.676)
Lesion							
Unilateral	746 (86.14)	0.82±0.19	0.37±0.29	0.64±0.21	0.52±0.25	0.50±0.25	0.45±0.25
Binaural	120 (13.86)	0.82±0.17	0.38±0.32	0.64±0.20	0.55±0.25	0.53±0.23	0.45±0.27
t (p)		−0.373 (0.709)	−0.449 (0.653)	−0.017 (0.987)	−1.434 (0.152)	−1.315 (0.189)	0.311 (0.756)
History of taking ototoxic drugs							
Yes	6 (0.69)	0.91±0.04	0.47±0.43	0.60±0.15	0.53±0.37	0.61±0.38	0.41±0.38
No	860 (99.31)	0.82±0.19	0.37±0.30	0.64±0.21	0.52±0.25	0.50±0.24	0.45±0.25
t (p)		1.163 (0.245)	0.785 (0.433)	−0.526 (0.599)	0.133 (0.894)	1.067 (0.286)	−0.394 (0.693)
Current degree of hearing loss							
Normal hearing (with tinnitus, dizziness and other symptoms)	85 (9.82)	0.85±0.15	0.40±0.36	0.63±0.19	0.55±0.27	0.53±0.29	0.49±0.29
Mild, 26–40 dB	319 (36.84)	0.82±0.18	0.36±0.29	0.66±0.20	0.51±0.25	0.49±0.24	0.46±0.25
Moderate, 41–60 dB	293 (33.83)	0.82±0.18	0.36±0.29	0.63±0.22	0.51±0.24	0.48±0.24	0.43±0.25
Severe, 61–80 dB	102 (11.78)	0.80±0.22	0.40±0.30	0.62±0.20	0.52±0.25	0.52±0.24	0.45±0.25
Extremely severe, >80 dB	67 (7.73)	0.78±0.20	0.41±0.25	0.67±0.20	0.57±0.22	0.54±0.22	0.48±0.22
F (p)		1.869 (0.114)	1.040 (0.385)	1.293 (0.271)	1.095 (0.358)	1.513 (0.196)	1.057 (0.377)

dB: decibel, a calculation unit for measuring the relative loudness of sound.

### Correlation analysis of coping style, social support and type D personality

The problem-solving and help-seeking dimensions were positively correlated with social support while the self-blame, fantasy and avoidance dimensions were negatively correlated with social support (p<0.05). The problem-solving and help-seeking dimensions were negatively correlated with type D personality while the self-blame, fantasy, avoidance and rationalization dimensions were positively correlated with type D personality (p<0.05). The strongest correlation was found between the self-blame dimension and type D personality, whereas the weakest was between the fantasy dimension and objective support. The specific correlation coefficients are shown in Table 3.

**Table 3.** Correlation analysis of coping style, social support and type D personality in young and middle-aged SSNHL patients (N=866)

	Social support				Type D personality	
Variables	Subjective support	Objective support	Utilization of social support	Total score	Negative affectivity	Social inhibition
Problem solving	0.230**	0.168**	0.129**	0.247**	−0.319**	−0.385*
Self-blame	−0.092**	−0.128**	−0.080*	−0.128**	0.346**	0.497**
Help seeking	0.158**	0.124**	0.274**	0.225**	−0.165**	−0.142**
Fantasy	−0.030	−0.071*	−0.016	−0.049	0.247**	0.351**
Avoidance	0.027	−0.100**	−0.023	−0.024	0.232**	0.313**
Rationalization	0.014	0.017	−0.051	0.001	0.197**	0.274**

* p<0.05, **p<0.01.

### Multiple linear stepwise regression analysis of factors related to coping style

The scores of various dimensions of coping style were used as dependent variables; meaningful indicators in univariate analysis and correlation analysis, including gender, chronic diseases, trauma history, social support (i.e. subjective support, objective support, utilization of social support, social support total score and social support level) and type D personality (i.e. negative affectivity and social inhibition), were used as independent variables. The inclusion criterion was set to 0.05 and the removal criterion was set to 0.10 and a multiple linear stepwise regression analysis was conducted. The multicollinearity test and residual analysis were used to assess the adequacy of regression models. Tolerance and variance inflation factor (VIF) results showed no collinearity (Table 4) and standardized residual histograms and normal probability plots showed that the results fit a normal distribution (Supplement Figure 1). The final results showed that gender, chronic diseases, trauma history, social support and type D personality were the factors related to the coping style of young and middle-aged SSNHL patients, which had different effects on each dimension of coping style (Table 4).

**Table 4.** Multiple linear stepwise regression analysis of related factors for coping style in young and middle-aged SSNHL patients (N=866)

Related factors	b	Sb	b′	t	p	Tolerance	VIF
Problem solving^a^							
Constant	0.861	0.064	–	13.408	<0.001	–	–
Subjective support	0.006	0.002	0.138	3.823	<0.001	0.735	1.360
Negative affectivity	−0.010	0.002	−0.283	−5.945	<0.001	0.421	2.375
Social inhibition	−0.004	0.002	−0.107	−2.419	0.016	0.489	2.044
Self-blame^b^							
Constant	0.261	0.096	–	2.727	0.007	–	–
Negative affectivity	0.018	0.003	0.316	8.954	<0.001	0.421	2.374
Type D personality	−1.000	0.029	−0.167	−3.496	<0.001	0.386	2.590
Help seeking^c^							
Constant	0.652	0.096	–	6.808	<0.001	–	–
Gender	0.030	0.014	0.073	2.199	0.028	0.982	1.019
History of trauma	−0.096	0.031	−0.102	−3.103	0.002	0.983	1.017
Utilization of social support	0.020	0.004	0.184	5.189	<0.001	0.849	1.178
Fantasy^d^							
Constant	0.617	0.102	–	6.019	<0.001	–	–
Gender	0.034	0.016	0.068	2.129	0.034	0.985	1.015
History of trauma	−0.095	0.036	−0.085	−2.651	0.008	0.986	1.014
Negative affectivity	0.010	0.002	0.204	4.172	<0.001	0.422	2.367
Type D personality	−0.062	0.026	−0.123	−2.404	0.016	0.388	2.577
Avoidance^e^							
Constant	0.436	0.074	–	5.856	<0.001	–	–
Negative affectivity	0.008	0.002	0.173	3.462	0.001	0.423	2.366
Social inhibition	0.005	0.002	0.100	2.210	0.027	0.508	1.967
Rationalization^f^							
Constant	0.667	0.091	–	7.304	<0.001	–	–
Chronic diseases	−0.037	0.017	−0.071	−2.178	0.030	0.982	1.018
History of trauma	−0.096	0.032	−0.097	−2.986	0.003	0.987	1.013
Negative affectivity	0.006	0.002	0.142	2.873	0.004	0.425	2.353
Type D personality	−0.073	0.023	−0.164	−3.169	0.002	0.388	2.578

^a^R^2^=0.183, ΔR^2^=0.176, F=27.436, p<0.001.

^b^R^2^=0.246, ΔR^2^=0.241, F=46.726, p<0.001.

^c^R^2^=0.082, ΔR^2^=0.073, F=9.534, p<0.001.

^d^R^2^=0.128, ΔR^2^=0.122, F=21.021, p<0.001.

^e^R^2^=0.096, ΔR^2^=0.092, F=22.861, p<0.001.

^f^R^2^=0.110, ΔR^2^=0.104, F=21.171, p<0.001.

## Discussion

At present, there are few studies on the coping styles of young and middle-aged SSNHL patients. The purpose of this study was to investigate the coping styles and related factors of these patients in China to provide references for clinical nursing practice. The results of this study showed that the ranking of average scores in the six dimensions of coping style were problem solving, help seeking, fantasy, avoidance, rationalization and self-blame. Gender, chronic diseases, trauma history, social support and type D personality may also affect the coping style of young and middle-aged SSNHL patients.

The coping styles are cognitive and behavioural methods or strategies that individuals adopt purposefully in order to reduce the impact of a specific stress.^20^ Generally speaking, problem solving, help seeking and rationalization are active and mature coping styles, while self-blame, avoidance and fantasy are negative and immature coping styles. Therefore the coping styles of young and middle-aged SSNHL patients in this study were mostly positive (high scores in the problem-solving and help-seeking dimensions). The study results showed that gender was a factor related to coping styles in this patient population and male patients had more positive coping styles, while female patients were more likely to adopt negative coping styles. The study by Kim et al.^21^ found that male patients were more inclined to actively seek social support to solve their difficulties than female patients, while female patients used active coping styles (e.g. confrontation and problem-solving coping styles) less than male patients, which is consistent with our results. It is largely because young and middle-aged female patients reported more stressful life events, such as separation problems (separation from parents or other relatives), physical illness or injury and pregnancy-related problems (unintended pregnancy or abortion), making women's coping styles more negative than those of men.^21^ So medical staff should pay more attention to timely psychological assessment and necessary psychological guidance of female patients in order to help them relieve the psychological stress caused by negative life events and encourage them to actively cope with difficulties.

In addition, our study indicated that patients with chronic diseases use more proactive coping styles. A study of long-term haemodialysis patients found that some patients realized the importance of life during the long-term treatment of their disease. As a result, they developed new attitudes and beliefs and actively coped with stressful situations.^22^ Active coping strategies can help individuals adapt to long-term and unpredictable stress.^22^ Some studies have shown that the coping styles of patients with chronic conditions are closely related to life satisfaction and disease perceptions.^23,24^ Therefore it is necessary to strengthen disease knowledge education and self-coping guidance for patients with chronic diseases who are ineffective at coping.

At present, only a limited number of studies have addressed the coping style of patients with trauma. According to the results of our study, the history of trauma had an impact on both the positive and negative coping styles of young and middle-aged SSNHL patients and patients with a trauma history were more likely to adopt positive coping styles. However, Birkeland et al.’s research^25^ found that people tend to think repeatedly about the injury process when dealing with traumatic events, and this ruminating coping style can make people develop frustrating thoughts about negative life events. Vaughn-Coaxum et al.’s study^26^ also mentioned that different degrees of trauma (including different trauma types or durations) may be associated with a specific coping style related to the risk for mental health problems. A longer duration of trauma or cumulative exposure to trauma was associated with more use of negative coping styles such as avoidance. Therefore, for SSNHL patients with a trauma history, medical staff need to assess the extent and characteristics of the injury, understand the different coping styles and psychological states of each patient and provide targeted psychological care.

Subjective support is obtained through emotional experience, such as being understood and accepted, and other support closely related to subjective feelings; objective support is an indispensable resource to meet people's physiological and social needs, including material and group relationship assistance. Utilization of support is the degree of utilization of support when encountering difficulties.^27^ In our study, subjective support for young and middle-aged SSNHL patients scored the highest, while patients’ utilization of support was low and the overall level of social support was not high. One study suggested that positive coping styles use direct self-improvement or external support to solve the predicament, while negative coping styles mostly reduce the impact of negative events through compromise or evasion, but usually the problem cannot be effectively solved.^28^ These studies suggest that it is equally important for patients to be proactive in using the support they receive to cope with difficulties.

Our study results indicate that social support is positively correlated with positive coping styles and negatively correlated with negative coping styles. Patients with higher levels of social support had more positive coping styles to face their disease. In addition, Tang and Dai’s research^29^ pointed out that coping style is an intermediary factor between social support and psychological distress, and an active coping style promotes the protective effect of social support on mental health. A high level of social support and positive coping styles reduce the risk of depression^29^ and coping styles and social support have a certain predictive effect on anxiety.^30^ Therefore, for young and middle-aged SSNHL patients who are prone to adverse psychological problems, medical staff should pay more attention to the role of social support in coping styles and psychological states in future clinical nursing practice.

The results of our study showed that type D personality is negatively correlated with positive coping styles and positively correlated with negative coping styles. On the whole, type D personality promoted negative coping and negative coping styles contributed to negative experiences such as social inhibition and negative affectivity. Our findings were consistent with those of Ginting et al.,^15^ that type D personality is associated with more passive coping styles of resignation or withdrawal. O’Riordan et al.’s study^31^ also found that people with type D personality had a lower level of social support in social interactions, so they had more negative perceptions of negative emotions and coping styles. Young and middle-aged SSNHL patients are prone to mental and psychological disorders,^32^ so SSNHL patients with type D personality are more likely to adopt negative coping styles, which is not conducive to recovery. Moreover, nearly half of the participants in this study were type D personality. Therefore it is advisable that medical staff conduct psychological and personality screening of SSNHL patients and provide psychological behaviour intervention for patients with type D personality to change their negative coping styles.

In view of the results of this study, in young and middle-aged SSNHL patients, medical staff should pay attention to the psychological evaluation of female patients and patients with chronic diseases or a trauma history and strengthen the screening of patients with type D personality to determine the factors affecting the coping style of these patients as soon as possible and take effective action. It is also necessary to help patients gain adequate social support and encourage them to utilize the support they receive to actively cope with the disease.

The sample size of this study was 866 patients. If a two-tailed test was used, assuming that the probability of type I error (α) is 0.01, and setting the effect size to 0.2, the test power (1−β) could reach 0.9995; i.e. the probability of type II error was extremely small. Nevertheless, this study also has certain limitations. Due to the limitations of research conditions, this study only recruited participants in Suzhou City and conducted a cross-sectional survey of young and middle-aged hospitalized patients, so the representativeness of the findings is limited. In the future, the sample size and survey scope can be expanded and stratified random sampling methods can be adopted to explore the coping styles of SSNHL patients in different regions, different age groups and different disease stages. Moreover, further longitudinal or interventional studies can be carried out to expand the study on coping styles and related factors in this patient population and form a complete nursing intervention guidance plan.

## Conclusions

This study preliminarily determined that gender, chronic diseases and a history of trauma may influence the coping style of young and middle-aged SSNHL patients, the level of social support is positively correlated with the positive coping style and type D personality has a negative impact on the coping style. It is advised that medical staff pay attention to the psychological assessment of female patients and patients with chronic diseases or a trauma history, strengthen the screening of patients with type D personality and promote patients’ social support, so as to help patients adopt active coping styles.

## Supplementary Material

ihac046_Supplemental_File

## Data Availability

The authors have full control of all primary data and agree to allow the journal to review the data if requested.

## References

[1] Plontke SK. Diagnostics and therapy of sudden hearing loss. GMS Curr Top Otorhinolaryngol Head Neck Surg. 2018;16:doc05. 29503670 10.3205/cto000144 PMC5818684

[2] Chandrasekhar SS , Tsai Do BS, Schwartz SR et al. Clinical practice guideline: sudden hearing loss (update). Otolaryngol Head Neck Surg. 2019;161(1 suppl):S1–S45. 31369359 10.1177/0194599819859885

[3] Schreiber BE , Agrup C, Haskard DO et al. Sudden sensorineural hearing loss. Lancet. 2010;375(9721):1203–11. 20362815 10.1016/S0140-6736(09)62071-7

[4] Ciesla K , Lewandowska M, Skarzynski H. Health-related quality of life and mental distress in patients with partial deafness: preliminary findings. Eur Arch Otorhinolaryngol. 2016;273(3):767–76. 26242252 10.1007/s00405-015-3713-7 PMC4762916

[5] Beutler LE , Harwood TM, Kimpara S et al. Coping style. J Clin Psychol. 2011;67(2):176–83. 21136534 10.1002/jclp.20752

[6] Veisani Y , Jalilian Z, Sadeghifard YZ et al. Association between common stressful life events and coping strategies in adults. J Educ Health Promot. 2021;10:307. 34667807 10.4103/jehp.jehp_519_20 PMC8459853

[7] Geoffrion S , Goncalves J, Robichaud I et al. Systematic review and metaanalysis on acute stress disorder: rates following different types of traumatic events. Trauma Violence Abuse. 2022;23(1):213–23. 32588756 10.1177/1524838020933844

[8] Yuan Y , Wang H, Chen QY et al. Illness experience and coping styles of young and middle-aged patients with sudden sensorineural hearing loss: a qualitative study. BMC Health Serv Res. 2021;21:742. 34315453 10.1186/s12913-021-06763-z PMC8314487

[9] Pössel P , Burton SM, Cauley B et al. Associations between social support from family, friends, and teachers and depressive symptoms in adolescents. J Youth Adolesc. 2018;47(2):398–412. 28695369 10.1007/s10964-017-0712-6

[10] Zhou Y , MacGeorge EL, Myrick JG. Mental health and its predictors during the early months of the COVID-19 pandemic experience in the United States. Int J Environ Res Public Health. 2020;17(17):6315. 32877985 10.3390/ijerph17176315 PMC7503583

[11] Escobar D , Noll P, Jesus TF et al. Assessing the mental health of Brazilian students involved in risky behaviors. Int J Environ Res Public Health. 2020;17(10):3647. 32455911 10.3390/ijerph17103647 PMC7277166

[12] Huang Y , Su X, Si M et al. The impacts of coping style and perceived social support on the mental health of undergraduate students during the early phases of the COVID-19 pandemic in China: a multicenter survey. BMC Psychiatry. 2021;21:530. 34706690 10.1186/s12888-021-03546-y PMC8549419

[13] Wlodarczyk D. Optimism and hope as predictors of subjective health in post-myocardial infarction patients: a comparison of the role of coping strategies. J Health Psychol. 2017;22(3):336–46. 26324234 10.1177/1359105315603470

[14] Borkoles E , Kaiseler M, Evans A et al. Type D personality, stress, coping and performance on a novel sport task. PLoS One. 2018;13(4):e196692. 10.1371/journal.pone.0196692 PMC5919645 29698480

[15] Ginting H , van de Ven M, Becker ES et al. Type D personality is associated with health behaviors and perceived social support in individuals with coronary heart disease. J Health Psychol. 2016;21(5):727–37. 24934433 10.1177/1359105314536750

[16] Sun ZQ , Xu YY. Medical statistics. Beijing, China: People's Health Publishing; 2014.

[17] Song RJ. Application of psychological capital in the treatment of depression. Shanxi, China: Shanxi Medical University; 2014.

[18] Xiao SY. The theoretical basis and research application of “Social Support Rating Scale”. J Clin Psychiatry. 1994;4(2):98–100.

[19] Reng ZT. Study on the correlation between newly diagnosed type 2 diabetes and type D personality in Xining area. Xining, China: Qinghai University: 2019.

[20] Fteiha M , Awwad N. Emotional intelligence and its relationship with stress coping style. Health Psychol Open. 2020;7(2):401950737. 10.1177/2055102920970416 PMC7656878 33224513

[21] Kim JE , Song IH, Lee S. Gender differences of stressful life events, coping style, symptom severity, and health-related quality of life in patients with panic disorder. J Nerv Ment Dis. 2017;205(9):714–9. 28609311 10.1097/NMD.0000000000000696

[22] Ghaffari M , Morowatisharifabad MA, Mehrabi Y et al. What are the hemodialysis patients’ style in coping with stress? A directed content analysis. Int J Community Based Nurs Midwifery. 2019;7(4):309–18. 31641680 10.30476/IJCBNM.2019.81324.0 PMC6779919

[23] García Montes JM , Sánchez Elena MJ, Valverde Romera M. The influence of coping and personality styles on satisfaction with life in patients with chronic kidney disease. Psychol Belg. 2020;60(1):73–85. 32166039 10.5334/pb.518 PMC7059424

[24] Cheng C , Yang CY, Inder K et al. Illness perceptions, coping strategies, and quality of life in people with multiple chronic conditions. J Nurs Scholarsh. 2020;52(2):145–54. 32017388 10.1111/jnu.12540

[25] Birkeland MS , Blix I, Thoresen S. Trauma in the third decade: ruminative coping, social relationships and posttraumatic stress symptoms. J Affect Disord. 2021;278:601–6. 33035947 10.1016/j.jad.2020.09.095

[26] Vaughn-Coaxum RA , Wang Y, Kiely J et al. Associations between trauma type, timing, and accumulation on current coping behaviors in adolescents: results from a large, population-based sample. J Youth Adolesc. 2018;47(4):842–58. 28555292 10.1007/s10964-017-0693-5 PMC6171358

[27] Shao R , He P, Ling B et al. Prevalence of depression and anxiety and correlations between depression, anxiety, family functioning, social support and coping styles among Chinese medical students. BMC Psychol. 2020;8:38. 32321593 10.1186/s40359-020-00402-8 PMC7178943

[28] Haskell AM , Britton PC, Servatius RJ. Toward an assessment of escape/avoidance coping in depression. Behav Brain Res. 2020;381:112363. 31739002 10.1016/j.bbr.2019.112363

[29] Tang W , Dai Q. Depressive symptoms among first-year Chinese undergraduates: the roles of socio-demographics, coping style, and social support. Psychiatry Res. 2018;270:89–96. 30245381 10.1016/j.psychres.2018.09.027

[30] Liu Q , Mo L, Huang X et al. Path analysis of the effects of social support, self-efficacy, and coping style on psychological stress in children with malignant tumor during treatment. Medicine (Baltimore). 2020;99(43):e22888. 33120834 10.1097/MD.0000000000022888 PMC7581179

[31] O'Riordan A , Howard S, Gallagher S. Type D personality and life event stress: the mediating effects of social support and negative social relationships. Anxiety Stress Coping. 2020;33(4):452–65. 32223435 10.1080/10615806.2020.1746284

[32] Yin Y , Lu Q. Effects of life events and emotional stress on short-term curative efficacy in adolescents with sudden hearing loss. J Clin Otorhinolaryngol Head Neck Surg. 2020;34(3):255–8. 10.13201/j.issn.2096-7993.2020.03.017 PMC10127865 32791595

