# Supplementary material for: Related factors to the coping style of patients with sudden sensorineural hearing loss
Source: Int Health. 2022 Jul 18;15(2):198–206. doi: 10.1093/inthealth/ihac046 (PMC9977213; doi:10.1093/inthealth/ihac046)
Supplement: ihac046_Supplemental_File [file ihac046_supplemental_file.docx]

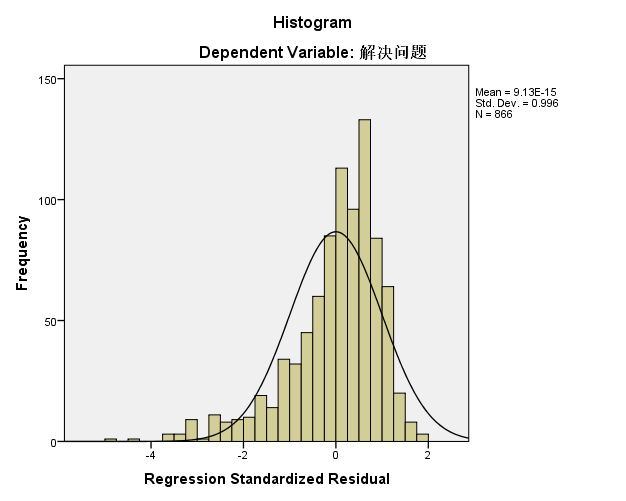

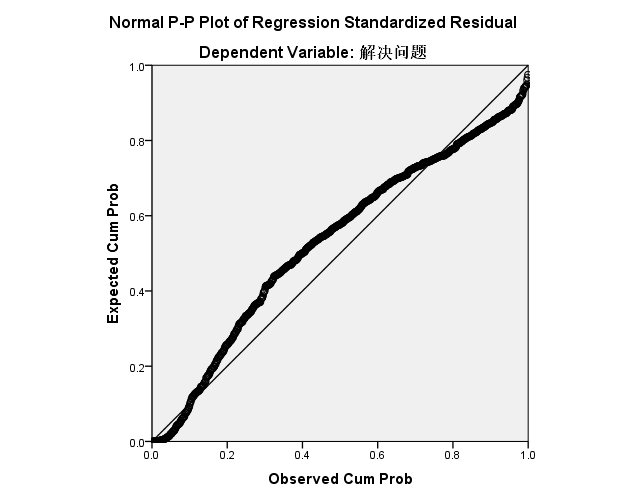


Standardized residual histogram P-P plot

a


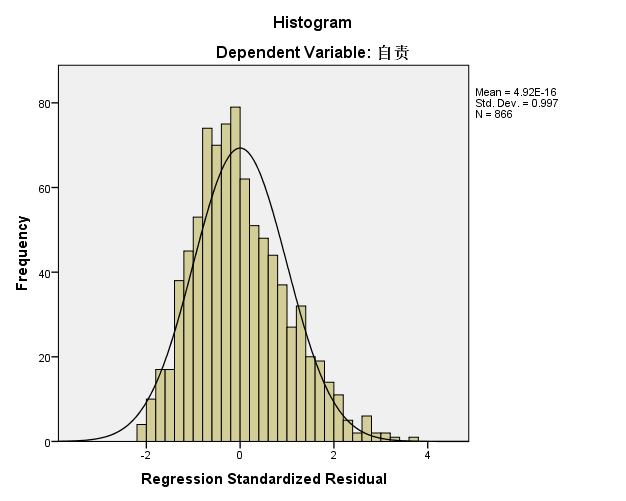

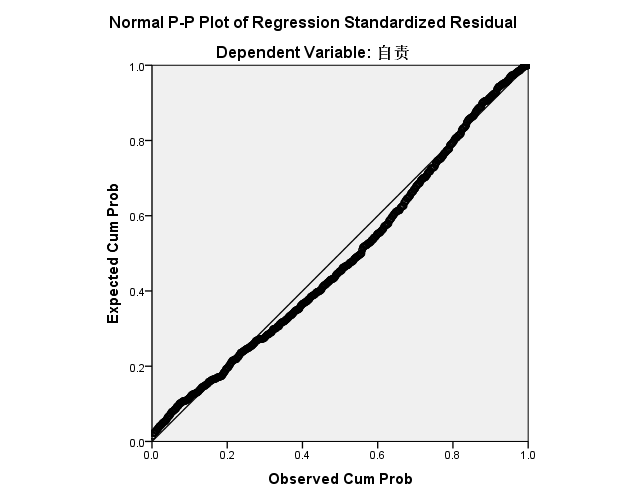


Standardized residual histogram P-P plot

b


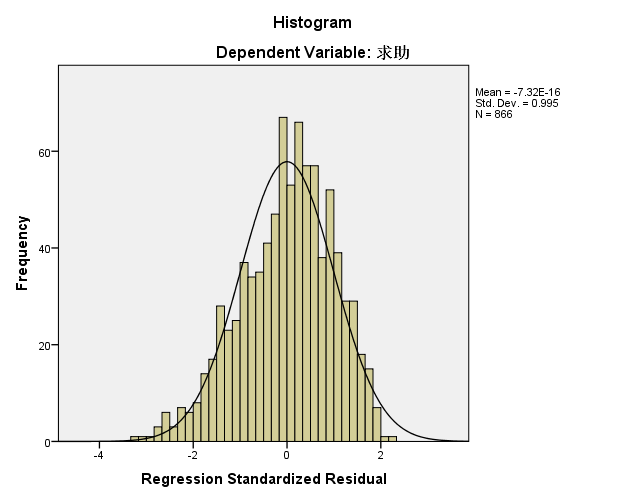

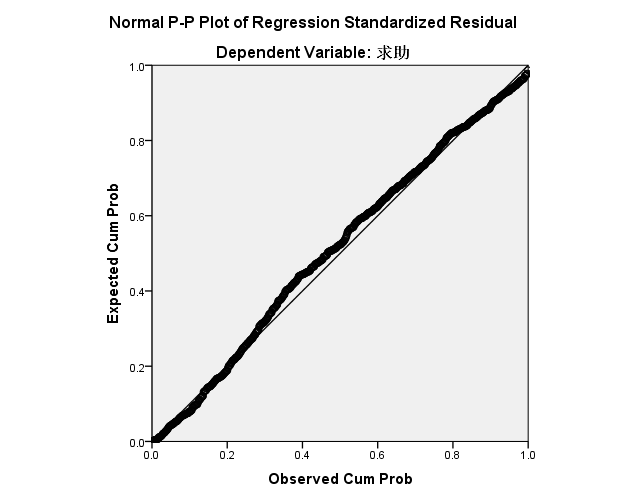


Standardized residual histogram P-P plot

c


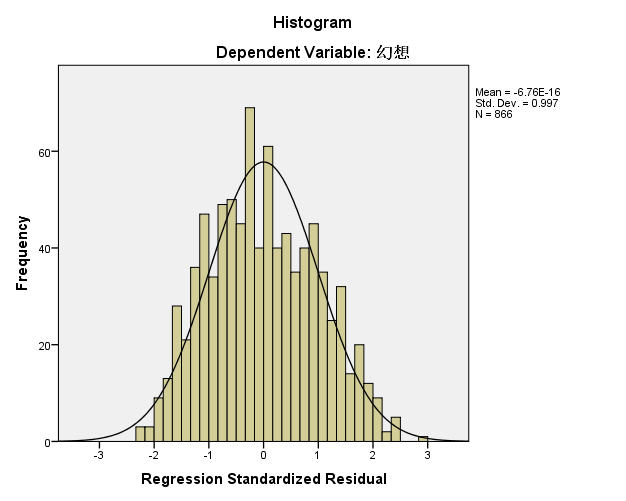

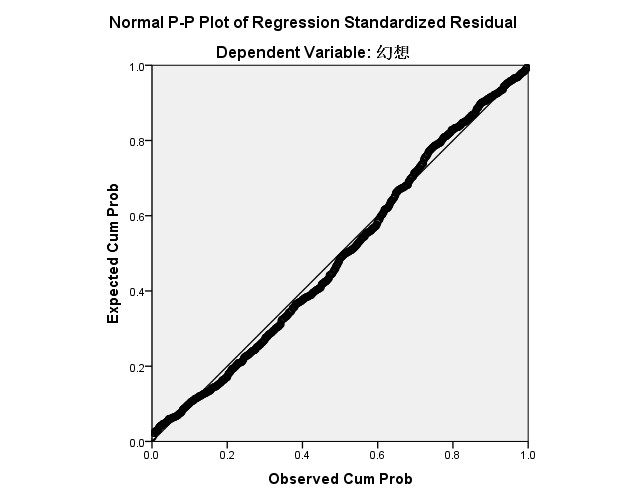


Standardized residual histogram P-P plot

d


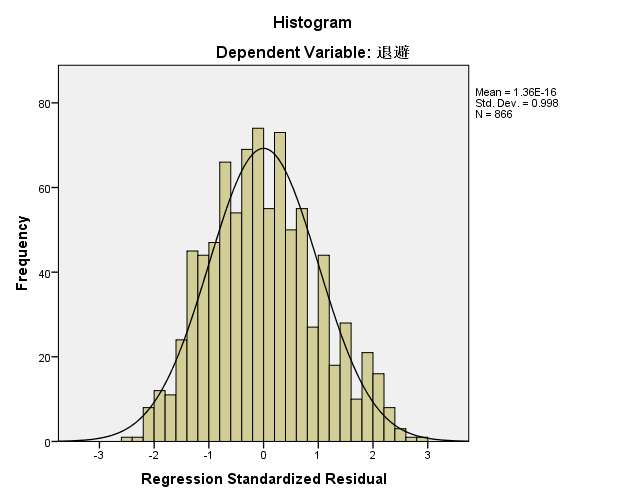

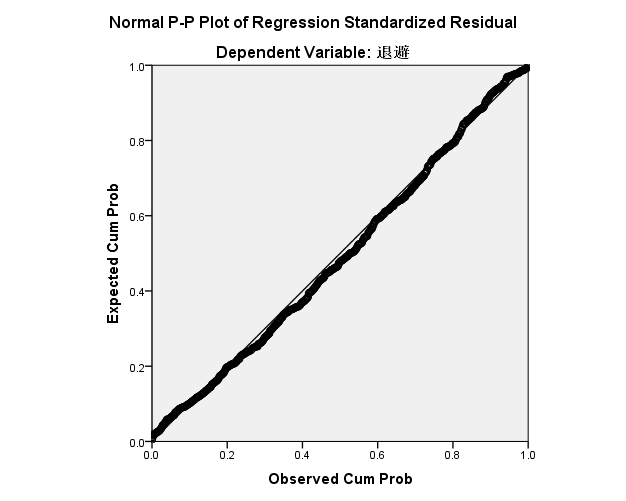


Standardized residual histogram P-P plot

e


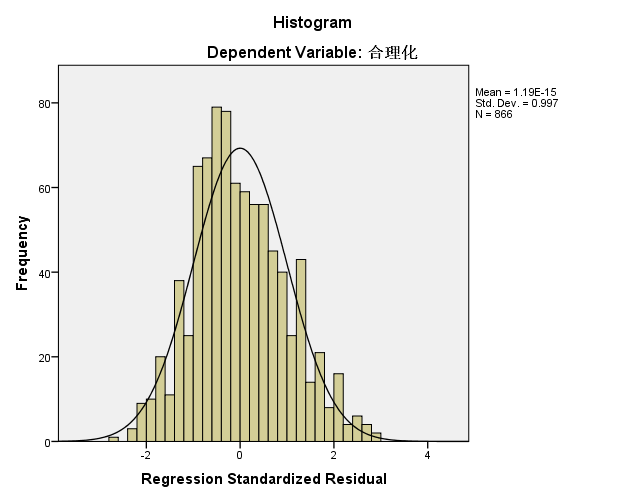

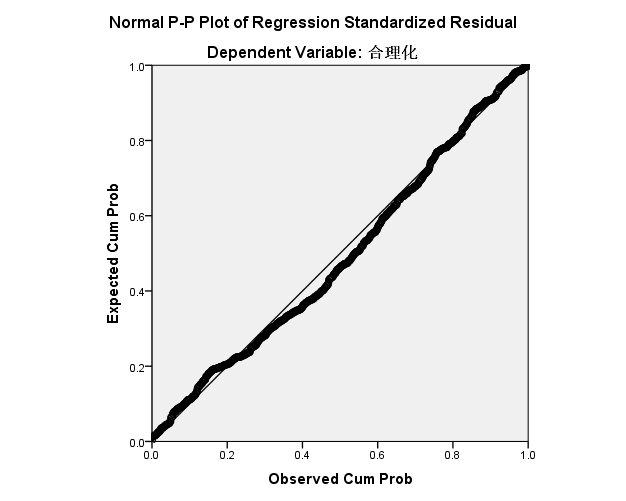


Standardized residual histogram P-P plot

f

Supplement Fig.1 The normality test of the residuals

Note: P-P plot: Normal probability plot; a: Problem-solving dimension; b: Self-blame dimension; c: Help-seeking dimension; d: Fantasy dimension; e: Avoidance dimension; f: Rationalization dimension.
